# Synaptic Potentiation Facilitates Memory-like Attractor Dynamics in Cultured *In Vitro* Hippocampal Networks

**DOI:** 10.1371/journal.pone.0057144

**Published:** 2013-03-20

**Authors:** Mark Niedringhaus, Xin Chen, Katherine Conant, Rhonda Dzakpasu

**Affiliations:** 1 Interdisciplinary Program in Neuroscience, Georgetown University Medical Center, Washington, District of Columbia, United States of America; 2 Department of Physics, Georgetown University, Washington, District of Columbia, United States of America; 3 Department of Neuroscience, Georgetown University Medical Center, Washington, District of Columbia, United States of America; 4 Department of Pharmacology and Physiology, Georgetown University Medical Center, Washington, District of Columbia, United States of America; SUNY Downstate MC, United States of America

## Abstract

Collective rhythmic dynamics from neurons is vital for cognitive functions such as memory formation but how neurons self-organize to produce such activity is not well understood. Attractor-based computational models have been successfully implemented as a theoretical framework for memory storage in networks of neurons. Additionally, activity-dependent modification of synaptic transmission is thought to be the physiological basis of learning and memory. The goal of this study is to demonstrate that using a pharmacological treatment that has been shown to increase synaptic strength within *in vitro* networks of hippocampal neurons follows the dynamical postulates theorized by attractor models. We use a grid of extracellular electrodes to study changes in network activity after this perturbation and show that there is a persistent increase in overall spiking and bursting activity after treatment. This increase in activity appears to recruit more “errant” spikes into bursts. Phase plots indicate a conserved activity pattern suggesting that a synaptic potentiation perturbation to the attractor leaves it unchanged. Lastly, we construct a computational model to demonstrate that these synaptic perturbations can account for the dynamical changes seen within the network.

## Introduction

A major focus in dynamical neuroscience is identifying the neural patterns of activity that characterize human behavior as well as its surroundings. For example, it is thought that organized network activity in the form of synchronized depolarization is critical to cognitive processes such as attention and memory consolidation [1], [2], [3], [4], [5], [6]. How neurons code the diversity of features in the environment and the assessment of the dynamic range of temporal responses when presented with external stimuli are some of the fundamental questions currently under investigation. However, an equally important question is how neurons self-organize into clusters or assemblies of coherent activity. These clusters of neural activity are thought to represent patterns that define different features within the external environment and the cluster constituents might change to reflect different environmental elements such as color and shading. While it is thought that the timing between neurons or neural assemblies is involved, how neural signals cluster or self-organize within a circuit to retrieve information that is related to a particular sensory stimulus or simply to recall a long-term memory is largely unknown. It is thought that a stimulus-dependent persistence in neural activity underlies active, i.e., working memory [7], [8], [9], [10] and was first postulated by Hebb [7]. This persistent activity is thought to be the result of strong reciprocal or recurrent excitatory connections between co-active neurons. The self-organization of activity displayed by neurons in these types of recurrent circuits accounts for the ‘delay between stimulation and response, that seems so characteristic of thought’ [7].

This neural correlate of memory has been incorporated into computational models of memory and it is believed that the dynamical correlate of working memory is the attractor state [8], [11], [12], [13], [14], [15]. An attractor is a stable dynamical pattern of activity to which a system evolves over time [12], [16], [17]. When the system is slightly perturbed, it will continue to evolve towards the attractor. Attractors have been widely used to model memory states because the dynamics of attractors are self-sustaining, i.e., they exhibit persistent activity in the absence of external stimulation [13]. Attractor models consist of recurrently connected networks of neurons via excitatory synapses, the connection between neurons, to reflect what was hypothesized by Hebb. When the network is presented with an external pattern, this pattern is stored via the modification of the recurrent excitatory synapses and results in a persistent increase in firing rates [18]. As experimental studies support the presence of attractors *in vivo* during hippocampal-dependent memory tasks [19], [20], this led us to ask whether similar patterns of activity might be retained in networks of hippocampal neurons in the absence of an intact anatomical architecture. Our experiments assess the impact on network dynamics after applying a pharmacological treatment that modulates the strength of excitatory synapses.

Network activity can evoke changes in the density of the 2-amino-3-(5-methyl-3-oxo-1, 2- oxazol-4-yl) propanoic acid (AMPA) glutamate receptor subunits that are present on the spines found in excitatory synapses [21], [28]. Such perturbations can influence action potential probability and the resulting firing rate within a network of neurons. These types of synaptic modulations have been observed in association with learning and memory and are thought to underlie the neural substrate of memory known as long-term potentiation, LTP [22], [23], [24], [25], [26], [27]. LTP results from the increase in synaptic efficacy between neurons and can be induced via high frequency electrical stimulation between pairs of neurons, or chemical stimulation and has been shown to last from several hours to many days [30], [31]. If a population of neurons is subjected to this modification, they can self-organize and cluster into active assemblies of elevated activity. If this activity persists, these assemblies might exhibit attractor dynamics.

LTP has been well studied between pairs of neurons within the hippocampus, specifically on synapses between the Schaffer collateral axons and apical dendrites of the CA1 pyramidal neurons [29], [33], [34]. However the impact on network dynamics due to the synaptic modifications modulated by LTP protocols has not been widely studied in experimental systems. In addition, computational models have successfully incorporated the attractor paradigm as a mechanism through which information storage can be reliable invoked. Therefore, the goal of our experiments and computational modeling is to assess whether a synaptic perturbation that is thought to underlie the physiological basis of memory is characterized on the network level by the theoretical postulates of memory.

Consequently, this paper reports on the temporal network activity that arises when a pharmacological paradigm of LTP-chemical LTP – is introduced in cultured hippocampal neurons. Chemical LTP is a method to induce potentiation of neurons without direct synaptic stimulation [35], [36], [37]. When applied to cultured networks, the need for electrical stimulation is eliminated. Chemical LTP has been shown to activate various biochemical pathways, such as increasing the concentration of cAMP that in turn is believed to increase the AMPA receptor density and is a useful technique to manipulate potentiation in large neural populations such as cultured networks. A cocktail of two drugs are used to induce chemical LTP. Forskolin activates adenylyl cyclase, and rolipram is a phosphodiesterase inhibitor. Together, this cocktail increases the levels of cyclic AMP thereby potentiating a large fraction of synapses in the network [36]. This results in an increase in the probability of neuronal spike generation. These separately performed experiments are consistent with our prior observations that were recorded at a later time point showing chemical LTP dependent effects on firing rate and increased bursting [38]. This current study focuses on an earlier time point and, along with more sophisticated analyses, tests the hypothesis that these *in vitro* results facilitate memory-like attractor dynamics. Additionally, we constructed a computational model consisting of biologically plausible neurons found in the hippocampus to assess whether manipulation of AMPA receptor density can account for the dynamical effects recorded in the experiments.

Experimentally, we use an array of extracellular electrodes, a multi-electrode array (MEA), to record spontaneous electrical activity when networks of hippocampal neurons have been pharmacologically perturbed. MEAs have been widely used to characterize dynamical activity from *in vitro* networks of neurons [39], [40], [41], [42]. In addition, MEA studies that implement electrical stimulation protocols on *in vitro* networks of either hippocampal or cortical neurons have been established demonstrating precedence for an *in vitro* learning paradigm [43], [44], [45], [46], [47], [48]. Lastly, an important temporal pattern found within developing *in vivo* circuits is the widespread prevalence of bursting activity [49], [50], [51]. Bursts are important during development as they facilitate normal functioning in developing neurons that in turn helps to create viable connections. We use young networks of cultured hippocampal neurons to study how a chemical LTP paradigm modulates network activity. We study network interactions at a time when the dynamics display a rich mix of vigorous bursting and spiking activity suggesting that these early periods are when the competition between spikes and bursts is at maximal levels. In our experiments, we show that network-wide firing rates increase but the variability in inter-spike intervals decrease. In addition, we show that the bursting frequency dramatically increases after chemical LTP evoking an elevation of network activity reminiscent of attractor dynamics. Our computational model shows that increasing AMPA receptor density can account for the increased epochs of network activity seen after chemical LTP. Furthermore, we show that increasing this receptor density on the pyramidal neuron population plays a predominant role. Therefore, our results suggest that the molecular modulations at the synapse, stimulated by the increased potentiation, results in the restructuring of the bursts as they form tightly compacted epochs of persistent activity, which may be indicative of an attractor basin of memory formation within the neural circuit.

## Materials and Methods

### A. Cell Cultures

#### Ethics Statement

All experimental procedures were approved by the Georgetown University Animal Care and Use Committee (GUACUC). Hippocampal tissue was extracted from embryonic day 18 Sprague-Dawley rats using a protocol modified from [52]. Briefly, the neural tissue was finely chopped and digested with 0.1% trypsin followed by mechanical trituration. Upon reaching a single cell suspension, 200,000 cells were added to multi-electrode arrays (MEA, Multi Channel Systems MCS GmbH, Reutlingen, Germany) that were previously treated with poly-d-lysine and laminin (Sigma, St. Louis, MO) resulting in an approximate density of 600 cells/mm^2^. Cells were counted using a hemacytometer (Hausser Scientific, Horsham, PA) and Trypan Blue (Sigma, St. Louis MO) was used to exclude non-viable cells. Cultures were maintained in Neuralbasal A medium with B27, penicillin/streptomycin and fetal bovine serum (Invitrogen, Carlsbad, CA) with bi-weekly changes and kept in a humidified 5% CO_2_ and 95% O_2_ incubator at 37°C.

### B. Electrophysiological recordings

We recorded all spontaneous electrical activity using a multi-electrode array. This MEA is composed of 59 titanium nitride electrodes, one reference electrode and four auxiliary analog channels each of which is 30 µm in diameter, arranged on an 8×8 square array. The inter-electrode spacing is 200 µm. Upon plating, the cells in suspension adhere to the silicon nitride substrate of the MEA and after seven days spontaneous electrical activity is detectable. We use the MEA1060 preamplifier and sample electrical activity at a 10 kHz acquisition rate in order to allow the detection of multi-unit spikes. The data was digitized and stored on a Dell personal computer (Round Rock, TX). Possible exposure to contaminants and fluctuations in osmolality and pH were significantly reduced during the data acquisition period by the use of an MEA cover made of a hydrophobic membrane [53]. This membrane provides a tight seal, is semi-permeable to CO_2_ and O_2_ and is largely impermeable to water vapor. Experiments from at least three MEAs for each condition, including controls, were performed on a heated stage at 37°C for at least 45 minutes at 14 days *in vitro* (14DIV), a time point during development in which the network displayed vigorous spontaneous electrical activity and for which network connectivity is well-established [54]. To ensure reproducibility of results across animals, all reported experimental groups were comprised of multiple cultures derived from multiple experimental preparations. Results obtained from cultures within and across different preparations were not significantly different.

### C. Pharmacological Induction of LTP

We used the pharmacological agents forskolin (50 µM) and rolipram (100 nM) to induce chemical LTP. Forskolin was dissolved in dimethyl sulfoxide (DMSO) to a stock concentration of 50 mM. Rolipram was dissolved in DMSO to a stock concentration of 100 µM. Both chemicals and DMSO were acquired from Sigma-Aldrich (St. Louis, MO).

We applied this chemical LTP treatment to the cultured hippocampal neurons on 14DIV. Initially, baseline electrical activity was recorded for 20 minutes on a heated stage at 37°C. To induce chemical LTP, 100 µL of conditioned media, the media in which the cells are continually maintained, was first removed from the MEA. Into this conditioned media, 1 µL of each stock solution of forskolin and rolipram was diluted. The treated media was then slowly added back into the MEA. MEAs were returned to the stage and recordings resumed immediately lasting for at least 30 minutes. Results are presented for the period 20 minutes after recording.

To control for possible solvent effects as well as mechanical artifacts arising from the exchange of solutions, a series of MEA recordings were performed on cultures in which 1 µL of DMSO was diluted into the conditioned media of another set of cultures prior to returning it to the MEA. Neither forskolin nor rolipram were added to these MEAs (vehicle experiments).

### D. Data Analysis

We removed low frequency components by high-pass filtering all traces at 200 Hz. Extracellularly recorded spikes, i.e., downward voltage deflections from baseline, were detected using a threshold algorithm from Offline Sorter (Plexon Inc., Dallas TX), which was calculated as a multiple of the standard deviation 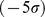 of the biological noise. We made no attempt to discriminate and sort spikes by electrode since the shape of a spike changes significantly during a burst due to changes in membrane excitability. In addition, for this study we concentrate on network activity and the signal from each electrode suitably reflects these dynamics.

We used custom software written in MATLAB (The MathWorks, Natick, MA) to analyze dynamical activity in the cultured hippocampal networks. To investigate changes in overall network activity, we calculated the average firing rate, FR, over a binned (10-second binsize), five-minute window for each electrode within an MEA. Values are reported as averages ±SEM. We then calculated the ratio of firing rates after treatment with respect to baseline for both the chemical LTP experiments and the vehicle. Next, to obtain a measure of spiking regularity, we calculated the coefficient of variation, CV, defined as the following: 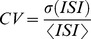



where 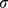 is the standard deviation of the inter-spike interval (ISI) distribution.

**Figure 1:**
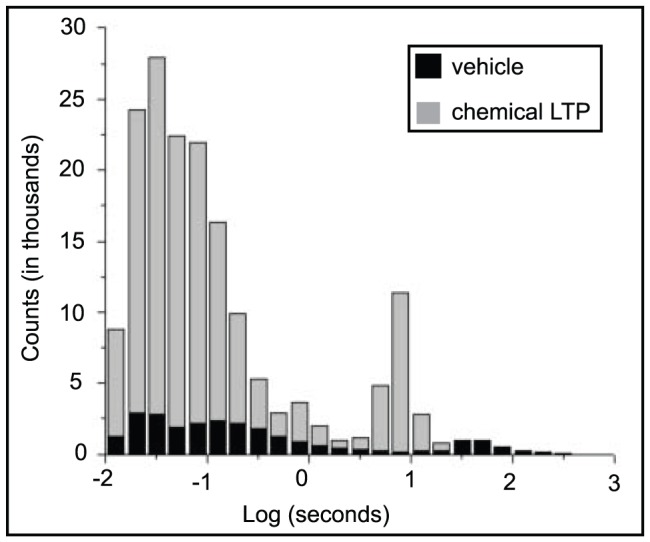
Log histograms of inter-spike intervals (ISI) display a bimodal distribution after chemical LTP (grey bars). The first peak is clustered around short ISIs – this defines the intervals within the bursts whereas the second peak is near 10 second and corresponds to the interval between bursts.

Next, we investigated changes in a common temporal feature found in cultured networks, the burst, as it represents a collective network response. In our experiments, we analyzed bursts from each individual electrode. After the spike detection process described above each electrode has a resulting spike train, 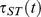, expressed as:
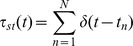
where N is defined to be the total number of spikes, 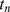 is the time of the *n*th spike and 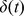
is a delta function that indicates a spike taking place at time 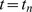. The inter-spike interval between spike *n* and spike *n*−1 (n>1) is: 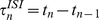
For both the control and chemical LTP experiments, we define a burst from each electrode to consist of no less than four spikes with a maximum inter-spike interval (ISI) of 100 ms. Log histograms of the ISIs indicated that this corresponded to the cutoff of the first peak (fig. 1–described below) in both conditions. Lastly, the burst durations, 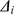, are defined to be:
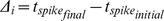



The final result of the burst identification process resulted in an M×N matrix where M corresponds to the electrode number and N's are the time stamps of the spikes within the bursts.

Lastly, we generated return maps of voltage activity to investigate the presence of nonlinear dynamical structures in the envelope of each bursting episode before and after chemical LTP treatment. The objective was to investigate the presence of a preserved structuring of the burst profile after chemical LTP. As with the initial assessment of the bursts, i.e., number of bursts and durations of bursts, we start with the high-pass filtered voltage signal from each electrode in order to exclude low frequency, field potential components. Since we were interested in studying how the shape or profile of the bursts evolves, we then low-pass filtered (10 Hz) each electrode. This removes the details, i.e., spikes, within the burst and leaves the envelope of the burst. From this time trace we plotted V_i,t_ vs. V_i,t+1_ where V*_i_* is the voltage corresponding to electrode *i* at time, *t*. A regularly repeating motif suggests the presence of a conserved activity pattern.

### E. Computational Model

**Figure 2:**
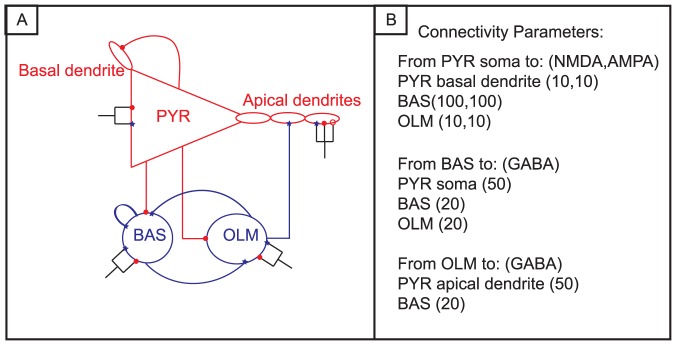
Schematic for computation model. The red triangle represents the population of excitatory pyramidal cells (PYR); blue circles are the inhibitory basket (BAS) cell population and oriens-lacunosum moleculare (OLM) cell population. Cells are randomly connected within each cell population. Truncated lines represent sites in which the synaptic weights were modified: red filled circles-AMPA, red open circles – NMDA and blue filled stars–GABA_A_. Numbers of AMPA, NMDA and GABA synaptic connections are listed in panel B.

To investigate whether the trafficking of AMPA receptors to the synapse can account for our observed network-wide effect, we used the simulator NEURON [55] to model the dynamics of a two-dimensional network consisting of 1000 biologically plausible neurons. We incorporated three cell types into the model that are believed to reflect the dynamics of hippocampal neurons (numbers of cells are parenthetically indicated): an excitatory pyramidal cell with simplified dendritic morphology (800), and two types of GABAergic interneurons: oriens-lacunosum moleculare (OLM; 100) and basket cells (BAS; 100) [56], [57], [58], 59. The pyramidal cell consists of five compartments: three apical dendrites, one basal dendrite coupled to the soma and both the basket and OLM cells were single compartment models. Cells are randomly connected within each type and these clustered homogeneous populations are connected to each other according to the diagram and connectivity schema in figure 2. There were a total of 124,000 synapses and they were randomly activated using a Poisson distribution. Synaptic and background activity parameters were taken from [59].

## Results

### A. Experimental

**Figure 3:**
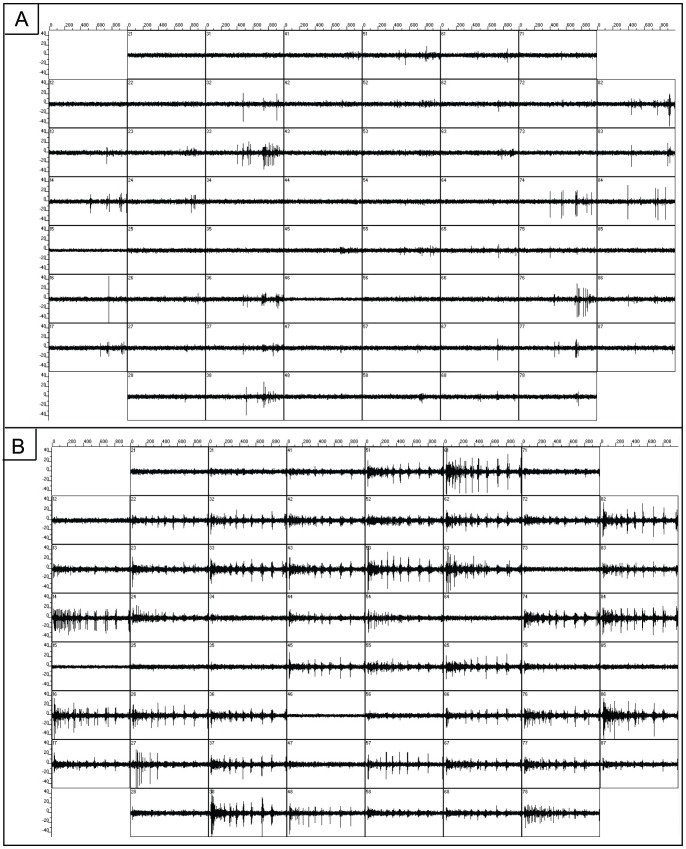
Spontaneous activity on an MEA before and after chemical LTP treatment. Each box represents one second (x-axis) of recording from one electrode and the voltage scale (y-axis) ranges from ±50 mV. A) Screen shot of spontaneous, high-pass filtered recording of baseline activity from an MEA. B) Screen shot of spontaneous, high-pass filtered recording of activity after chemical LTP performed on the MEA from panel A. In general, approximately 66% of the electrodes on each MEA displayed activity after chemical LTP (N = 4).

Fig. 3 is a representative screenshot of spontaneous, high-pass filtered activity as recorded by the MEA. Each box corresponds to one second of activity from one electrode. Each electrode records activity from neurons in its vicinity and a majority of these electrodes reveal robust activity. Panel A corresponds to baseline activity from one MEA and panel B is activity after treatment of chemical LTP from the same MEA. This image shows that is a large increase in spiking and bursting activity after chemical LTP and will be further quantified below.

**Figure 4:**
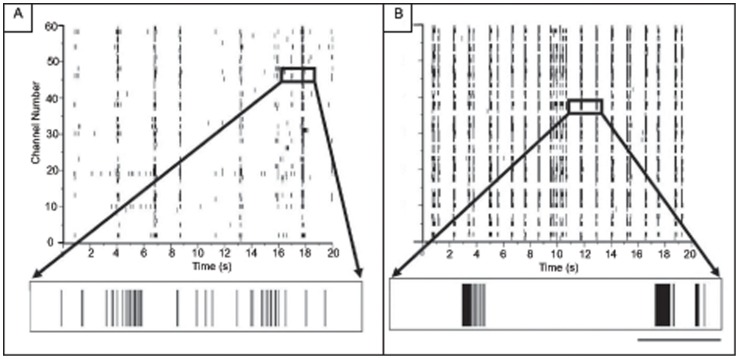
Network spiking activity is increased after chemical LTP treatment. A) Raster plots of 20 seconds of spontaneous activity at 14 days *in vitro* from untreated cultured hippocampal networks. There is a large degree of activity with each electrode displaying bursting and spiking dynamics. The duration of the burst shown in the expanded view is 300 ms. B) Raster plots 20 minutes after application of chemical LTP. The expanded views show that the bursts increase in frequency and appear to shorten in duration to an average of 81±12 ms. Scale bar = 100 ms.

Next, we created raster plots to highlight activity from all channels over a longer temporal scale. Fig. 4 presents raster plots of spiking activity over a 20-second time window from the control hippocampal networks (fig. 4A) and the hippocampal networks 20 minutes after the application of chemical LTP (fig. 4B). One row in each panel corresponds to one electrode and in each row each small vertical tick mark is a detected spike. Below each raster plot is an expanded view of activity that shows a mix of bursts and single spikes. The raw voltage trace from a selected electrode is presented at the bottom. The control network exhibits bursts of a long duration. After chemical LTP, the bursts appear to cluster into tightly organized episodes of shortened duration and higher frequency.

**Figure 5:**
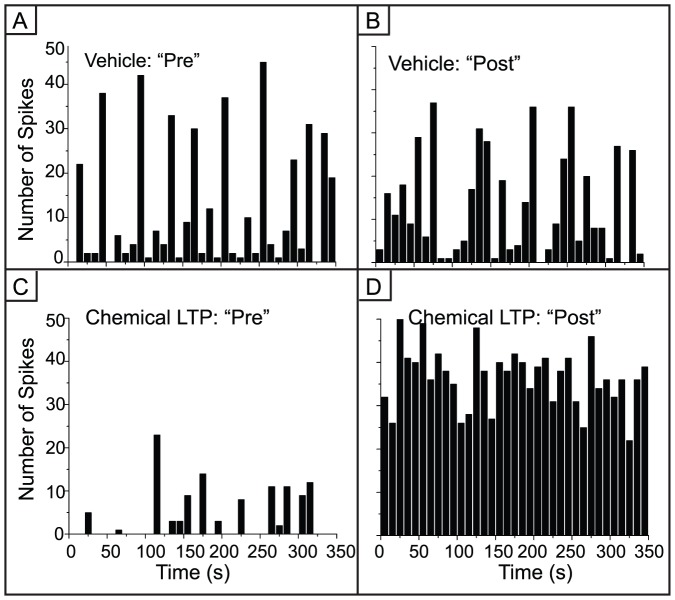
Variability in spiking activity is reduced after chemical LTP. A,B) Spike count histograms from a representative electrode in the vehicle networks. There is robust but highly variable spiking activity. C) Spike count histogram in an electrode before chemical LTP. D) While the initial baseline is low in this example (C), the spike rate increases dramatically after chemical LTP treatment and the variability is low (one-way ANOVA, p<10^−9^).

We began our analysis by investigating changes in overall network activity. Fig. 1 is a log histogram of the inter-spike intervals from the chemical LTP and vehicle experiments showing that there is considerably more activity after chemical LTP. In addition to the large increase in activity, there is a leftward shift in the distribution. Within the short interval regime, usually corresponding to the spike intervals within bursts, is a well-defined peak around 5 ms embedded within a log normal-like distribution. In the longer interval regime there is a singular, pronounced peak near 10 seconds, an interval associated with being between bursts. The average ratio of firing rates (firing rate ratio after treatment relative to baseline) across the vehicle MEAs was 1.96±0.73 whereas the average ratio for the chemical LTP MEAs was 6.19±2.25 (one-way ANOVA, p<10^−9^). The vehicle increase might be attributed to mechanical perturbations. Fig. 5 highlights these differences in a spike count histogram using representative electrodes from the vehicle and chemical LTP treatments. There is an increase in spiking activity in the chemical LTP electrode while the activity in the electrode from the vehicle culture remains largely unchanged.

**Figure 6:**
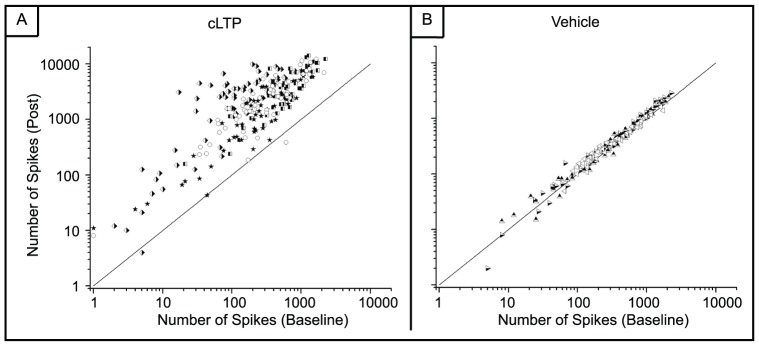
There is a persistent increase in spiking activity after chemical LTP. A) Spike counts from all electrodes before and chemical LTP. Most electrodes have an increase in activity with a large cluster displaying an increase of at least two orders of magnitude. (one-way ANOVA, p<10^−9^) B) Spike counts from the DMSO-treated MEAs show no increase in activity. (one-way ANOVA, p<10^−7^). Each symbol corresponds to a different MEA. Three MEAs were used for the vehicle and four MEAs were used for the chemical LTP studies. The diagonal line denotes y = x.

Next we looked at the relationship between the aggregate number of spikes within a five-minute window before and 20 minutes after chemical LTP or vehicle treatment. Electrodes from all MEAs within each treatment were pooled and their spike counts are displayed on a log scale (fig. 6). The diagonal line represents y = x and therefore points falling on this line have no change in activity. Nearly all of the electrodes from the chemical LTP MEAs are above this line indicating an increase in activity, with a majority showing an increase of more than two orders of magnitude (fig. 6A). MEA vehicle experiments showed negligible change in the number of spikes (fig. 6B).

**Figure 7:**
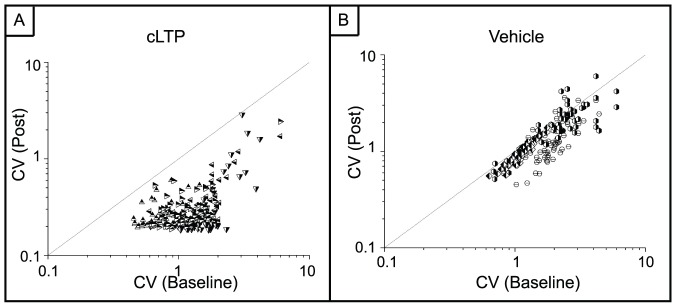
The coefficient of variation (CV) of inter-spike intervals is reduced after chemical LTP. A) CV from the chemical LTP MEAs. There is an overall reduction in the CV indicating that variability in activity has been reduced. (one-way ANOVA, p<10^−5^) B) MEAs treated with only DMSO show no change in the CV. (one-way ANOVA, p<10^−8^) Each symbol corresponds to a different MEA. Three MEAs were used for the vehicle and four MEAs were used for the chemical LTP studies. The diagonal line denotes y = x.

The profile of the time evolution of spiking activity in fig. 5 suggests that there is a change in the variability of inter-spike intervals (ISI) after chemical LTP. To address this, we calculated the coefficient of variation, CV, for all MEAs (fig. 7). There is a uniform decrease in the CV across all electrodes that experienced the chemical LTP treatment indicating that the variability in network activity was reduced. This reduction in CV for the chemical LTP networks is in sharp contrast to a negligible change for the vehicle MEAs (one-way ANOVA, p<10^−5^).

The increase in the firing rate and the decrease in variability of inter-spike intervals led us to ask how the chemical LTP treatment affected bursts, a subset of network activity. The burst, which is a tight barrage of spikes, is a dominant temporal motif in cultured networks, it is present in developing *in vivo* systems, and is believed to represent coordinated activity from neural assemblies [49], [50], [51]. It has been suggested that a burst may be more efficient to modulate information leaving a diminished role in information transmission for individual spikes [60], [61], [62]. If the bursts were positively impacted by the chemical LTP treatment, this would contribute to the increase in network regularity as seen in the reduction of the CV.

**Figure 8:**
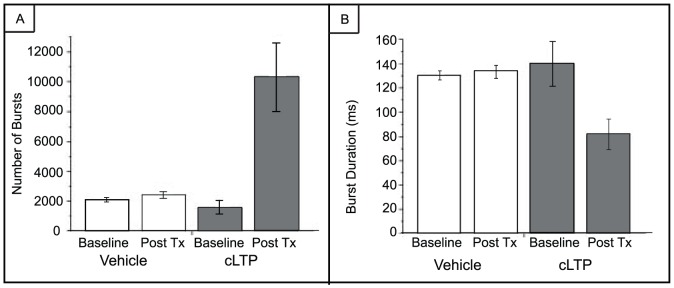
Number of bursts and burst durations of spontaneous and evoked activity. A) The bursting activity significantly increases (one-way ANOVA, p = 0.0003) after the application of chemical LTP, contributing to the overall increase in network firing rates as seen in fig. 4. B) The durations of the bursts decreases after chemical LTP (one-way ANOVA, p = 0.0004). Error bars represent SEM. Three MEAs were used for the vehicle and four MEAs were used for the chemical LTP studies.

Fig. 8 presents the number of bursts and burst durations from the chemical LTP and vehicle MEAs. Values are reported as averages ±SEM. There is a significant increase in the number of bursts after chemical LTP and this increase clearly contributes to the increase in the overall firing rate within the network as seen in the raster plots of figure 4. In the vehicle and pre-chemical LTP networks, the average number of bursts was approximately 2058±148 and 1564±429, respectively. However, the post-vehicle treatment increased the average number of bursts to approximately 2438±208 whereas 20 minutes after chemical LTP the average number of bursts increased to 10,300±2363 (one-way ANOVA, p = 0.0003). In addition, the burst durations decreased considerably after chemical LTP (fig. 8B). The average burst duration for the pre-chemical LTP MEAs was 140±18 ms and after treatment, 81±12 ms whereas the vehicle treatment the average was 130±3 ms before and 133±5 ms after treatment (one-way ANOVA, p = 0.0004). This decrease in event duration suggests that the collective network activity contracted and experienced a re-organization into short episodes.

Bursts represent the collective network response to our pharmacological perturbation and only those spikes that participate within a burst are considered in the burst analyses. The raster plots of figure 4 suggest that there may be a reduced number of spikes in between the bursts and therefore, we calculated the fraction of spikes not in bursts as a percent change from baseline. In the baselines of both the vehicle and chemical LTP experiment, approximately 20% of the spikes were not in bursts. However, there was a marked change after chemical LTP; this fraction decreased nearly 50% while the fraction in the vehicle fluctuated minimally. Chemical LTP appears to incorporate more of the “errant” spikes into bursts, leaving the inter-burst regions quiescent.

**Figure 9:**
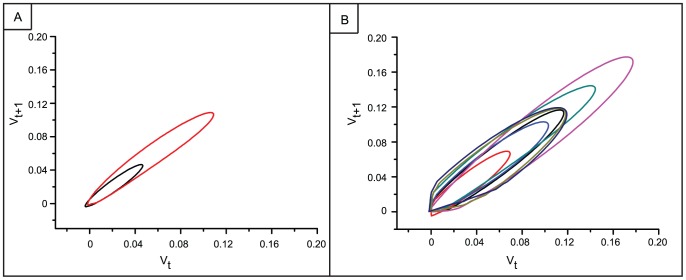
Conserved burst activity pattern is maintained after chemical LTP. A) Phase plot of bursts during 10 seconds of baseline activity. B) Phase plots of bursts during 10 second of activity after chemical LTP. Each motif repeats suggesting the preservation of an attractor state.

Lastly, fig. 9 presents a representative return map of the low-pass filtered voltage for 10 seconds of activity from an electrode before (fig. 9A) and after (fig. 9B) chemical LTP. These filtered traces represent the envelope of each bursting epoch of activity. Whenever there is a peak or trough in the envelope, the return plot will cross the identity line. Figure 9A shows that the baseline bursting activity pattern appears to be stable – each envelope shares a similar shape, an ellipse, and each ellipse represents the profile of one single burst. Within this time window, there were two bursts before treatment as reflected by the two ellipses in figure 9A. This elliptical shape is preserved after the chemical LTP treatment (fig 9B). The large increase in bursting activity after treatment is also reflected by the increase in the number of ellipses in figure 9B. The fact that the elliptical shape is preserved after the chemical LTP treatment suggests that synaptic potentiation conserves a spatiotemporal pattern of activity.

### B. Computational Model

**Figure 10:**
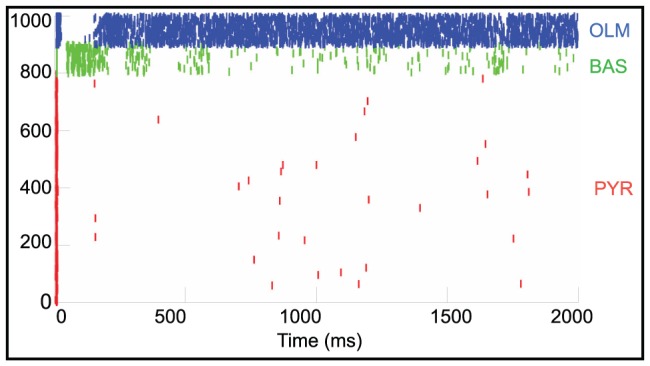
Basal network activity is sparse with only inhibitory OLM cells displaying high frequency spiking activity. Raster plot of simulated activity in which the red tick marks represents the excitatory pyramidal cells; green tick marks are inhibitory basket (BAS) cells and the blue tick marks are the inhibitory OLM cells.

While it has been shown that the trafficking of AMPA receptors to the synapse accounts for the biological mechanism underlying LTP on a small spatial scale [28], [31], [32], collective neural activity is not linear and we investigated whether the manipulation of AMPA receptors might account for our observed network-wide dynamical effects. Increasing AMPA receptor density can conceivably take two forms. The synaptic inputs could increase their strength or weight by increasing the number or density of AMPA receptor sites. In this model, the truncated lines represent the synaptic inputs, as a global parameter, as drawn in figure 2. Alternatively, the number of synaptic connections onto a given postsynaptic cell could increase while the overall density of synaptic inputs remains unchanged. This is represented by the connection from one cell to another in figure 2. Figure 10 is a raster plot of baseline network activity before we manipulated the presence of the AMPA receptors. The pyramidal cells are very sparsely active and only the inhibitory OLM cells have a large degree of spiking activity. Bursting activity had a frequency of 0.75±.02 Hz (one-way ANOVA, p = 0.0003).

**Figure 11:**
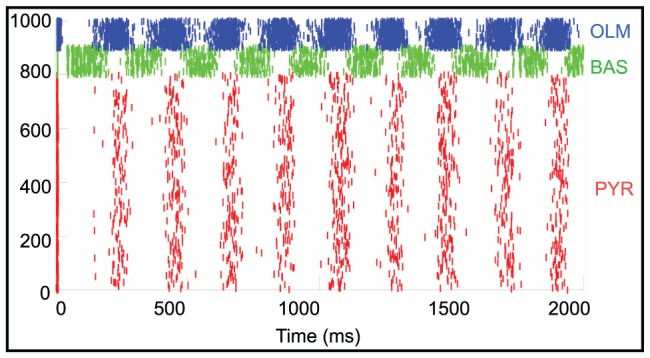
Synchronized bursting activity appears after synaptic weights have been increased. Raster plot of simulated network activity after AMPA synaptic weights were increased throughout the network. Red tick marks represents the excitatory pyramidal cells; green tick marks are inhibitory basket cells and the blue tick marks are the inhibitory OLM cells.

Next, we increased the AMPA synaptic weights at different cell sites that contain AMPA synapses according to figure 2. Figure 11 is a raster plot of network activity after a 30% increase in the synaptic weights of the pyramidal, basket and OLM cells' somas as well as at the apical dendrite of the pyramidal cell. Each cell population displays an increase in spiking activity and the activity is organized into bursting epochs. Bursting activity had a frequency of 4.7±1.3 Hz. This persistent spiking activity was very similar when we increased the AMPA strength by 30% at both the pyramidal cell soma and apical dendrite or only at the pyramidal cell soma while leaving the AMPA weights at control levels on the OLM and basket cell soma sites (data not shown).

**Figure 12:**
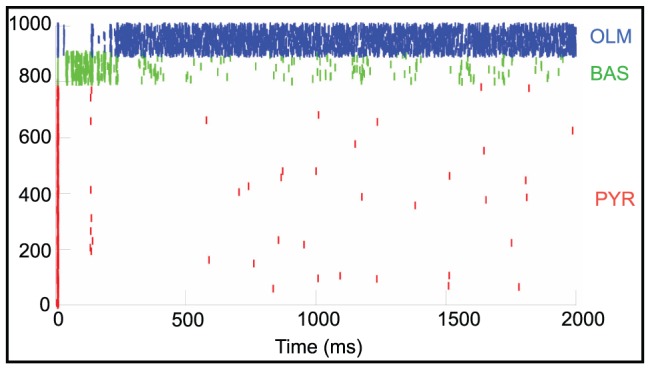
Increasing the number of AMPA connections does not result in synchronized bursting activity. Raster plot of network activity after AMPA connections were increased throughout the network. Red tick marks represents the excitatory pyramidal cells; green tick marks are inhibitory basket cells and the blue tick marks are the inhibitory OLM cells.

However, when only the apical dendrite AMPA weights were increased or when the soma sites of both basket and OLM cells were increased, leaving the pyramidal cell soma parameters unchanged, spiking activity did not increase (data not shown). Lastly, we varied the total number of AMPA connections at each cell population (fig. 12). Spiking activity did not appreciably increase above the baseline.

## Discussion

In these studies, we perform a global, biological manipulation that is believed to preferentially target a subset of structures residing on a small spatial scale – excitatory synapses ending on spines. We investigate the resulting dynamical effects on a large spatial scale-the network of cultured hippocampal neurons. The synapse was treated with a pharmacological paradigm that is known to increase the probability of action potential firing and quantified changes in spiking activity reflecting the response from the network. This increased likelihood of firing is due to the Hebbian-like strengthening of synapses that might occur during the creation of a memory [26], [65], [66], [67]. In addition, using a computational network model, we show that AMPA receptor trafficking results in the generation of persistent bursting activity within the network.

While the application of this drug cocktail may effect changes on the microscopic level other than synaptic modification, our results strongly indicate that synaptic perturbations can account for the observed modifications on the macroscopic level – overall network spiking activity. We suggest there may be two phenomena that could explain these changes. The chemical LTP treatment elevates network activity but the state remains stable. There is a major increase in overall network activity, as seen in the network firing rates. This is due to the increase in potentiation of a large fraction of synapses. This persistent activity due to an increase in connection strength has been theoretically described using attractor models.

In addition, there is a reduction in the coefficient of variation, CV, after chemical LTP. This reduction in the CV implies that the variability in the inter-spike intervals from the electrodes is reduced. The firing pattern becomes relatively constant with no large fluctuations of high activity. Regulation of neural activity must be preserved to prevent extremes in neural output – either hyperexcitability, which can lead to neurotoxic or neuropathological conditions, or insufficient excitation, which can cause the neuron to cease firing altogether [63], [64], [68]. These regulatory mechanisms on the cellular level must also propagate to the network level in the form of circuit-stabilizing mechanisms and it has been suggested that appropriately modulated activity within a neural circuit could be maintained via the modulation of firing rates [63], [64]. There may be a tuning range of firing rates over which the neural circuit operates most effectively. While it is too early after the treatment to assess long-term regulation of activity, our results suggest that the process of chemical LTP may facilitate the reduced variability of firing rates in the short term.

All of the firing rates from the electrodes increased dramatically after the chemical LTP treatment. However, the relative increase was not uniform across all electrodes and may be indicative of the different developmental stages of the neurons. These differences may also affect the ability of each neuron to respond to a synapse-strengthening perturbation. There is a small fraction of electrodes that displayed at least an eight-fold increase in multi-unit firing rate activity after treatment. This effect is further emphasized by the log scale presentation of spike counts produced by each electrode. As previously stated, we did not spike sort the data from these experiments. With our relatively low plating density, we rarely saw more than one unit per electrode (analysis not shown). We therefore introduce a possible scenario with the understanding that targeted biochemical assays are necessary to confirm our hypothesis. Chemical LTP modulates the neuron via several mechanisms and it will be the integrated effect that produces an increase in network-wide spiking activity. We focus, in this case, on one of these mechanisms and suggest that some of the neurons with this large firing rate increase are glutamatergic, i.e., excitatory, neurons with immature spines that responded with a vigorous spine expansion under chemical LTP induction. The spine expansion caused the firing rates of those cells to “catch up” to those of glutamatergic neurons with presumably more developed spines. This brought the previously immature cells within the range of the firing rates of the rest of the network. As a result, it appears from the dynamics within the network that all of the neurons, regardless of their initial developmental phase, had similar firing rates after treatment. Therefore, a striking network dynamical effect has materialized after chemical LTP in the reduced spiking variability. Chemical LTP has a differential effect on the increase in firing rates on clusters of neural assemblies, and these clusters may represent different information storing units.

Bursting activity in the network also displayed dramatic changes after synaptic potentiation. There is an increase in burst frequency, and the individual bursts are of a shorter duration and the shape of the attractor describing the burst profile was an ellipse. We perturbed the attractor using chemical LTP. Despite the fact that burst durations decreased, the shape of the attractor remained the same. The additional spikes generated by strengthening of the synapses need not have contributed to bursting activity and could have simply raised the background level of single spikes. Interestingly, not only did the burst frequency increase but there also was a large reduction in the fraction of spikes that are not participating in bursts accompanied by the preservation of the attractor profile. The large increase in the inter-spike interval histogram combined with the reduction of the number of spikes that do not participate in bursts suggest that the previously “errant” spikes were either recruited into existing bursts or, more likely, created new bursts with a shortened duration. It has been speculated that bursts may be more efficient at information processing within a neural circuit [60], [61], [62]. In these experiments, processing efficiency may represent information storage. We observe a repeating spatiotemporal pattern in the burst envelope return maps before and after treatment. The peaks and troughs of the envelope determined where the return maps crossed the identity line and the resulting similarity in shape before and after chemical LTP suggests that the system maintains a stable state of activity despite the persistent increase in activity. We note that assessing the shape of the attractor was not the intended focus of the study. The question was whether the shape, regardless of its type, would change after this particular perturbation. Nevertheless, the fact that the shape was an ellipse is intriguing. Perturbations performed outside the scope of this study resulted in a non-elliptical shape after treatment (data not shown but see ref. 40 for return maps of cortical cultured networks) suggesting that the elliptical profile can indeed be modified. This is currently under further investigation. Lastly, the reduction in the coefficient of variation of inter-spike intervals suggests a more “regular” network temporal structure. These combined results demonstrate that synaptic potentiation evokes physiological events that restructure the burst profile. These restructured bursts represent the creation of a new functional entity that appears to facilitate information storage within the network.

Our computational model illustrates the importance of the contribution of AMPA receptor trafficking to the persistent state of increased activity. Increasing the synaptic strength appears to have the largest effect on the pyramidal cell rather than on the basket or OLM cells. This suggests that there may be a differential impact of AMPA receptor trafficking and will require further experimental investigations. Lastly, we show that increasing the AMPA synaptic strength rather than the number of AMPA connections plays the predominant role in the generation of our observed tightly compacted epochs of persistent activity. Given that we are assessing changes to overall network activity a short time after application of the treatment, it would not be expected that entirely new connections would be created within this time interval.

## Conclusions

In conclusion, our integrated results demonstrate that a synaptic perturbation can account for a profound change in network dynamics. We used a chemical paradigm that facilitates synaptic strengthening to stimulate specific changes in network activity from cultured hippocampal neurons that are similar to results obtained from attractor-based computational models that describe memory storage. Our computational model provides a dynamical mechanism for our observations by demonstrating the role of AMPA receptors in the creation of increased persistent activity.

An applied stimulus to a neural system will influence its output, the spike. We asked the question, “Does a perturbation known to facilitate synaptic potentiation manifest as a dynamical correlate of memory on the global network scale?” While future studies are required for validation, the presence *in vitro* attractor dynamics in the form of persistent elevated activity suggests that fundamental principles of neural self-organization might be retained in the absence of anatomy.
